# Advanced Management of Severe Adenomyosis in IVF: A Personalized Approach With Extended GnRH Agonist and Letrozole Therapy

**DOI:** 10.1155/crog/4150637

**Published:** 2026-03-09

**Authors:** Charline Fatemi, Jonalyn Edades, Ibrahim ElKhatib, Francisco Ruiz, Laura Marqueta Marques, Laura Melado

**Affiliations:** ^1^ College of Medicine, European School of Medicine, Frankfurt, Germany, medcol.mw; ^2^ Medical Department, ART Fertility Clinics Abu Dhabi, Abu Dhabi, UAE

**Keywords:** adenomyosis, aromatase inhibitors, euploid blastocyst, frozen embryo transfer, hormonal suppression

## Abstract

**Objective:**

The objective of this study is to evaluate the effectiveness of prolonged GnRH agonist (GnRH‐a) suppression combined with letrozole and intensive luteal phase support in a patient with severe adenomyosis undergoing frozen‐thawed embryo transfer (FET).

**Design:**

The study design was a case report.

**Setting:**

The setting was a tertiary referral in vitro fertilization (IVF) clinic.

**Subjects:**

The subject was a 41‐year‐old woman with a history of primary infertility with severe adenomyosis and endometriosis. Her partner presented with nonobstructive azoospermia.

**Exposure:**

Following micro‐TESE, ovarian stimulation, and ICSI, the patient received prolonged combined GnRH‐a and letrozole suppression before two attempts of single euploid frozen embryo transfer (eFET).

**Main Outcome Measures:**

The main outcome measure was pregnancy outcome.

**Results:**

The first eFET cycle with 3 months of suppression resulted in an early miscarriage at 7 weeks. In the second cycle, a prolonged 5‐month suppression led to a significant reduction of the uterine size, lower serum estradiol levels, and optimal endometrial preparation, achieving an ongoing pregnancy and delivery of a healthy baby at 36 weeks of gestation.

**Conclusion:**

This case supports the potential benefit of extended GnRH‐a and letrozole suppression with intensive luteal phase support for patients with severe adenomyosis, suggesting that individualized protocols may be beneficial and warrant further investigation in similar complex cases.

## 1. Introduction

Adenomyosis is a condition characterized by the presence of endometrial tissue within the myometrium. While one in three patients with adenomyosis is asymptomatic, others may experience a wide range of symptoms, including pelvic pain and infertility, with heavy menstrual bleeding being the most common [1]. Historically, most women were diagnosed between 40 and 50, with histopathology being the primary diagnostic tool. However, advances in pelvic imaging, such as magnetic resonance imaging (MRI) and enhanced transvaginal ultrasound (TVUS) imaging, have led to more frequent diagnoses, including in younger patients [2]. Despite these advancements, adenomyosis is still challenging to diagnose [3]. It is also important to remember that adenomyosis is a condition that often, but not always, coexists with endometriosis [4].

Adenomyosis can cause several changes in the endometrium, including inflammation, abnormal uterine contractility, and infertility, all of which may negatively affect fertility treatment outcomes. A recent meta‐analysis demonstrated a detrimental effect of adenomyosis on IVF outcomes, including lower clinical pregnancy and live birth rates, as well as increased miscarriage risk [5]. Hormonal suppression therapy prior to the endometrial preparation for frozen‐thawed embryo transfer (FET) appears to be the preferred treatment for these patients [6]. However, recent data suggest that GnRH agonist (GnRH‐a) suppression alone may not significantly improve IVF outcomes in women with adenomyosis [5, 6].

In the herein case, we present a 41‐year‐old patient with severe adenomyosis and endometriosis, managed pharmaceutically prior to FET. This case illustrates that GnRH‐a‐induced hypopituitarism, combined with the aromatase inhibitor letrozole to suppress estrogen production by adenomyotic lesions and intensive luteal support to address progesterone resistance associated with adenomyosis, led to a successful ongoing pregnancy.

## 2. Case Report

A 41‐year‐old woman, G0, with a BMI of 26.8 kg/m^2^, was referred to our center in 2021, seeking fertility treatment after 1 year of primary infertility and a diagnosis of severe adenomyosis and endometriosis. She had a regular 25‐day menstrual cycle with severe dysmenorrhea. Her surgical history included four laparoscopies: one for myomectomy (2011) and three for ovarian cystectomy and adhesiolysis due to Stage IV endometriosis (2014, 2019, and 2020). At her initial consultation, TVUS and abdominal ultrasound revealed a globally enlarged uterus not fully visible with vaginal ultrasound, reaching up to the hepatic area, with diffuse adenomyosis, with > 80% of the junctional zone affected and including a 7‐cm adenomyotic nodule in the posterior uterine wall (score > 9) (Figure 1) [7]. Additionally, she presented a frozen pelvis with both ovaries in a high position behind the uterus, leading to difficult visualization and bilateral endometriomas. Her antral follicle count was 7, and her AMH was 1.9 ng/mL.

Figure 1(a) Abdominal ultrasound image of the uterus before initiating the first suppression cycle with GnRH‐a and letrozole. The image reveals a globally enlarged uterus with diffuse adenomyosis, characterized by asymmetrical wall thickening, hyperechoic islands and cystic areas, and an irregular, disrupted junctional zone. An adenomyotic nodule is visible in the posterior uterine wall. (b) Transvaginal ultrasound taken one cycle after early miscarriage following the first eFET attempt.

**Figure:**
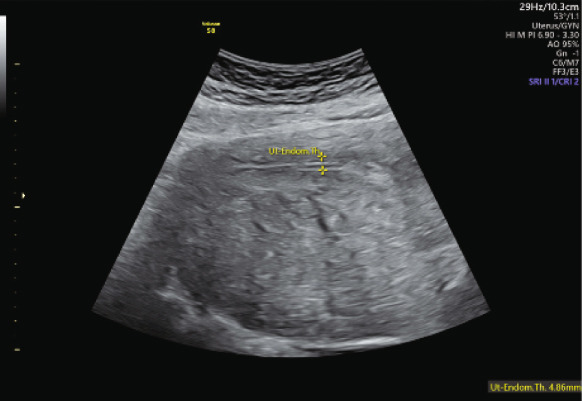
(a)

**Figure:**
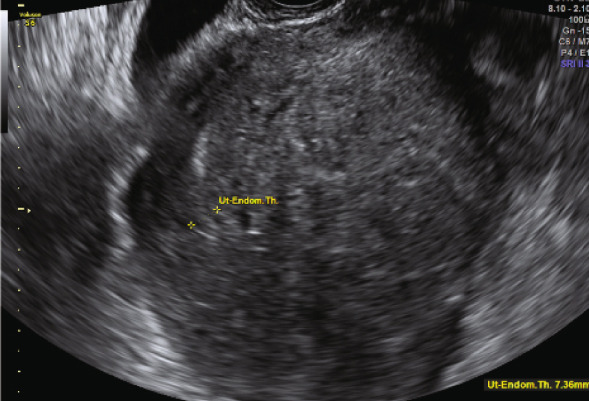
(b)

Her husband presented with nonobstructive azoospermia (NOA). His karyotype was normal (46XY), and no Yq microdeletions were observed. His hormonal profile showed FSH at 17.41 mIU/mL, LH at 7.45 mIU/mL, prolactin at 144.1 *μ*IU/mL, TSH at 1.15 *μ*IU/mL, and total testosterone at 469.8 nmol/L. He underwent bilateral microTESE in our center after 3 months of treatment with aromatase inhibitors (letrozole 2.5 mg daily) [8]. The sperm retrieved was cryopreserved in separate straws for future fertilization.

After counseling the couple on treatment options, they proceeded with ICSI, undergoing several ovarian stimulation cycles for oocyte retrieval and embryo accumulation due to age and the presence of NOA, which both are known to increase the risk of aneuploidy. A total of 12 blastocysts were generated, but only two were euploid following PGT‐A.

The first endometrial preparation for embryo transfer was planned after 3 months of a combination therapy with GnRH‐a depot (Decapeptyl 3.75 mg intramuscular monthly) and letrozole 5 mg/day. Hormonal evaluation prior to starting hormonal replacement therapy (HRT) showed estradiol < 5 pg/mL and progesterone < 0.05 ng/mL. Hysteroscopy was performed before the start of the HRT for endometrial preparation, with a vaginoscopy approach and uterine saline irrigation [9]. After 11 days of HRT with estradiol (6 mg/day orally), the endometrial thickness measured 9.6 mm on TVUS, with a triple‐line pattern. Luteal phase support was initiated with vaginal progesterone (Endometrin, 300 mg daily) and oral dydrogesterone (Duphaston, 30 mg daily) [10]. A Day 6 blastocyst (BB quality, BL7 expansion) was transferred, but this resulted in an early miscarriage at 7 weeks.

The second embryo transfer was planned using the same protocol (Decapeptyl 3.75 mg intramuscular monthly plus letrozole 5 mg/day orally); however, hormonal suppression was maintained for 5 months. Monthly TVUS assessments were performed to monitor uterine size and wall thickness until there was evidence of significant uterine shrinkage (Figure 2). Hysteroscopy for uterine saline irrigation was performed prior to starting HRT (Figure 3). Endometrial preparation combined oral estradiol (6 mg/day for 11 days) with vaginal progesterone (Utrogestan, 200 mg every 8 h) and intramuscular progesterone (100 mg every other day). The endometrial thickness before luteal phase support was 10.6 mm, again showing a triple‐line pattern. A Day 6 blastocyst was transferred (BB quality, BL7 expansion—indicating a fully expanded blastocyst with thinning of the zona pellucida, graded according to the Gardner and Schoolcraft system [11]) and resulted in an ongoing pregnancy. The patient successfully delivered a healthy baby via C‐section at 36 weeks.

Figure 2(a, b) Transvaginal ultrasound (TVUS) following endometrial preparation for the second eFET. The image shows notable uterine shrinkage compared to the image in Figure 1b, indicating a positive response to the prolonged suppression protocol.

**Figure:**
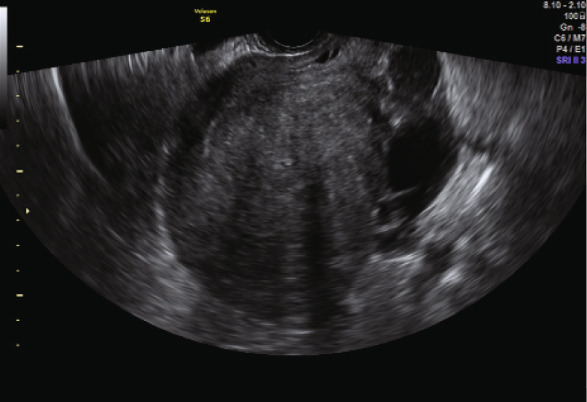
(a)

**Figure:**
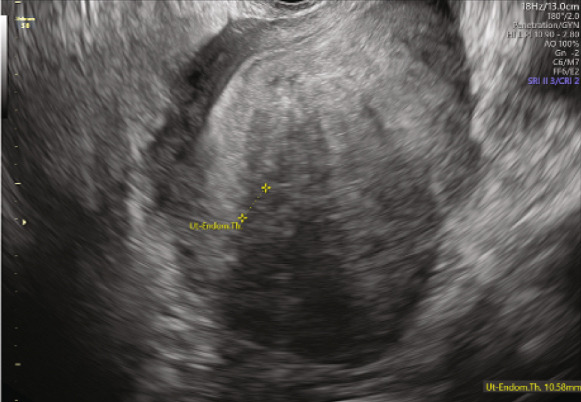
(b)

Figure 3Diagnostic hysteroscopy performed before starting the second endometrial preparation with HRT after 5 months of suppression with GnRH‐a and letrozole. (a) Note the elongated cervical canal and segment. (b) Fundus and the left wall of the uterine cavity. (c) Fundus and the right wall of the uterine cavity.

**Figure:**
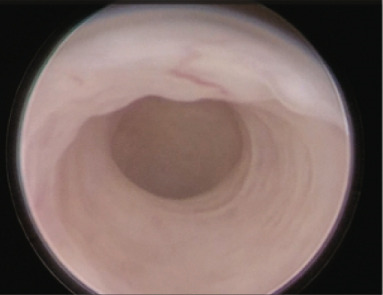
(a)

**Figure:**
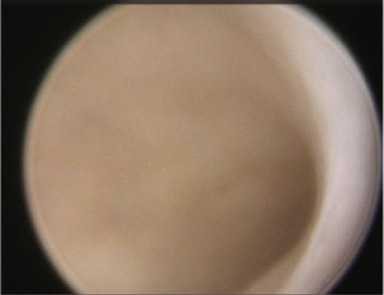
(b)

**Figure:**
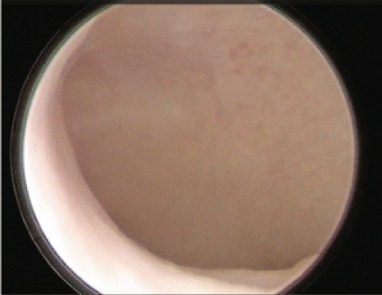
(c)

A comparison of the two eFET cycles is summarized in Table 1, highlighting differences in suppression duration, hormonal levels, luteal phase support, and outcomes.

**Table 1:** A comparison of both euploid frozen embryo transfer (eFET) cycles showed that the longer 5‐month suppression combined with intensified luteal phase support resulted in an ongoing pregnancy. TL, triple line; E2, estradiol; P4, progesterone; ET, embryo transfer.

	Suppression: Decapeptyl 3.75 im + letrozole 5 mg/day	Diagnostic hysteroscopy with saline instillation	Days of E2	Endometrial thickness	Serum E2 (before starting LPS)	Luteal phase support	Serum P4 day of ET	Blastocyst quality	bHCG levels	Outcome
1^st^ eFET	3 months	Yes	11 days	9.6 mm TL	125.40 pg/mL	Endometrin 300 mg/day + Duphaston 30 mg/day	8.25 ng/mL	Euploid, Day 6 BB, BL7	310 mIU/mL	Early miscarriage (7 weeks)
2^nd^ eFET	5 months	Yes	11 days	10.6 mm TL	115.9 pg/mL	Utrogestan 200 mg/8 h + progesterone 100 mg im/48 h	11.77 ng/mL	Euploid, Day 6 BB, BL7	370 mIU/mL	Live birth delivery at 36 weeks

## 3. Discussion

This case highlights the complexities of preparing the endometrium for FET in patients with severe adenomyosis. While recent studies suggest the potential benefits of combining GnRH‐as with aromatase inhibitors like letrozole, there remains limited literature on the optimal dosage, treatment duration, or specific patient populations that may benefit most. In this case, a prolonged combination of GnRH‐a and letrozole resulted in ultrasonographic evidence of significant uterine shrinkage, leading to a successful ongoing pregnancy and delivery of a healthy baby. The decision to extend suppression to 5 months was based on monitoring the signs of adenomyosis on monthly TVUS scans, based on both emerging clinical evidence [12] and the need for individualized protocol optimization in this complex case. Treatment was continued until there was clear ultrasonographic evidence of uterine shrinkage, guiding the timing of HRT initiation.

Previous studies demonstrated comparable efficacy of aromatase inhibitors and GnRH‐as in adenomyosis management [2]. However, due to an intrinsic aromatase activity, the adenomyotic tissue has the capability of autonomously producing estrogen [5, 6, 13]; hence, the most commonly used treatment, GnRH‐a downregulation, may not always reduce the local production of estradiol sufficiently. Cozzolino et al. [14] reported that even after five GnRH‐a injections, some patients maintained elevated serum estradiol concentrations. In this case, elevated estradiol levels were not due to peripheral estrogen conversion, as the patient was not obese. Combining GnRH‐a and letrozole effectively reduced serum estradiol levels (to < 5 pg/mL), in line with previous publications [14]. This highlights the benefit of close protocol monitoring for such patients, including (i) measuring FSH, LH, and estradiol levels to ensure adequate hormonal suppression and (ii) conducting regular TVUS evaluations to track uterine size, uterine wall thickness, and reduction in adenomyotic signs [12], thus allowing individualized treatment duration.

Before starting endometrial preparation with HRT, the index patient underwent office diagnostic hysteroscopy, with a vaginoscopy approach, for evaluation of the cervical canal and uterine cavity. This approach evaluates the uterine cavity, helps gather information about the cervical canal, and facilitates the lysis of cervical adhesions in preparation for the upcoming embryo transfer procedure. Additionally, other benefits from cavity irrigation have been hypothesized, like mechanically removing detrimental antiadhesive glycoproteins from the surface of the endometrium and initiating changes in the immune system and gene expression, subsequently improving the endometrial receptivity [9]. Recent studies have indicated that the hysteroscopy prior to embryo transfer might enhance the implantation due to the creation of an aseptic inflammation [9].

Progesterone resistance is an additional consideration in adenomyosis cases due to the downregulation mediated by the increased estrogen receptor expression in adenomyotic tissue [15, 16]. When planning a FET in patients with adenomyosis, intensive luteal phase support is necessary, together with close monitoring of the progesterone levels during the luteal phase and early pregnancy.

The combination of GnRH‐a and aromatase inhibitors implies significant side effects, such as severe menopausal symptoms, potentially leading some patients to discontinue treatment. Comprehensive patient management, including thorough counseling and support, is essential to help patients navigate these side effects. Additional risks of the long‐planned protocol, like the possible risk of osteoporosis, should be considered. Preventively, the index patient was put under calcium plus vitamin D supplementation.

A key strength of our case report is that both FET cycles were performed using high‐quality euploid embryos, eliminating aneuploidy as a contributing factor, especially relevant in the context of advanced maternal age and severe male infertility. Transferring euploid embryos in such challenging cases is crucial to reduce confounding variables that may influence implantation and pregnancy outcomes. However, using different luteal phase support regimens across the two cycles might represent a limitation. Furthermore, it should be noted that adenomyosis is independently associated with an increased risk of miscarriage, even when euploid embryos are transferred. Although suboptimal suppression was considered a contributing factor to the early miscarriage observed in the first cycle, other mechanisms cannot be excluded, including uterine contractility abnormalities, impaired endometrial receptivity, or immune dysregulation [12]. Notably, a recent prospective study by Cozzolino et al. [17] reported that adenomyosis, particularly when involving the junctional zone or when classified as severe, significantly increased the risk of miscarriage, despite the use of high‐quality donor oocytes and standardized endometrial preparation protocols. Additionally, it is important to highlight that in this case, the TVUS and abdominal ultrasound evaluations were particularly challenging due to the presence of a severely enlarged uterus, extending far above the umbilicus. This anatomical distortion necessitated the use of both transvaginal and abdominal routes to visualize the ovaries, which were located high behind the uterus and surrounded by adhesions. As a result, the AFC assessment was subject to significant variability and may have underestimated the true ovarian reserve. It is well recognized that AFC may be less reliable in cases of distorted pelvic anatomy, low ovarian accessibility, or the presence of large uterine pathology, and its predictive value may be inferior to that of serum AMH in such scenarios [18].

There are still important questions that remain unanswered. Firstly, there is a need to determine the appropriate treatment duration and dosing regimen, as the current guidelines do not adequately address this. Secondly, alternative approaches must be explored for patients who cannot tolerate this regimen. Finally, data on the potential impact of this treatment during the second and third trimesters of pregnancy remains scarce. Further research and establishing detailed protocols are essential to improve ART outcomes.

## 4. Conclusions

Complex adenomyosis cases like the one presented here, often involving challenging histories of infertility and/or obstetric complications, require individualized management. The combination of GnRH‐a and aromatase inhibitors with enhanced luteal phase support offers a promising treatment option for these patients. However, despite encouraging results, conclusions cannot be generalized based on a single case and should be interpreted cautiously. Further studies are required to confirm the efficacy of such an approach.

## Author Contributions

**Charline Fatemi**: conceptualization, validation, writing–original draft, writing–review and editing. **Jonalyn Edades**: conceptualization, data curation, project administration, validation, writing–review and editing. **Ibrahim ElKhatib**: methodology, resources, supervision, validation, writing–review and editing. **Francisco Ruiz:** conceptualization, data curation, methodology, resources, writing–review and editing. **Laura Marqueta Marques**: conceptualization, data curation, methodology, validation, writing–review and editing. **Laura Melado**: conceptualization, data curation, formal analysis, investigation, methodology, supervision, validation, visualization, writing–original draft, writing–review and editing.

## Funding

No funding was received for this manuscript.

## Disclosure

The subjects in this trial have not concomitantly been involved in other randomized trials (If applicable). Data regarding any of the subjects in the study has not been previously published unless specified.

## Consent

The patient included in this report gave consent for scientific publication, including social media, the journal website, scientific literature websites (e.g., PubMed, ScienceDirect, and Scopus), and other applicable sites.

## Conflicts of Interest

The authors declare no conflicts of interest.

## Data Availability

The data underlying this article cannot be shared publicly for the privacy of individuals who participated in the study. The data will be shared at a reasonable request by the corresponding author. Data will be made available to the journal editors for review or query upon request before and/or after publication.
